# Muscles Reduce Neuronal Information Load: Quantification of Control Effort in Biological vs. Robotic Pointing and Walking

**DOI:** 10.3389/frobt.2020.00077

**Published:** 2020-06-24

**Authors:** Daniel F. B. Haeufle, Isabell Wochner, David Holzmüller, Danny Driess, Michael Günther, Syn Schmitt

**Affiliations:** ^1^Hertie Institute for Clinical Brain Research, University of Tübingen, Tübingen, Germany; ^2^Center for Integrative Neuroscience, University of Tübingen, Tübingen, Germany; ^3^Institute for Modelling and Simulation of Biomechanical Systems, University of Stuttgart, Stuttgart, Germany; ^4^Stuttgart Center for Simulation Science, University of Stuttgart, Stuttgart, Germany; ^5^Machine Learning and Robotics Lab, University of Stuttgart, Stuttgart, Germany; ^6^Institute for Stochastics and Applications, University of Stuttgart, Stuttgart, Germany; ^7^Max-Planck Institute for Intelligent Systems, Stuttgart, Germany

**Keywords:** muscle, control effort, morphological computation, reinforcement leaning, reflexes during walking, information entropy, torque actuator

## Abstract

It is hypothesized that the nonlinear muscle characteristic of biomechanical systems simplify control in the sense that the information the nervous system has to process is reduced through off-loading computation to the morphological structure. It has been proposed to quantify the required information with an information-entropy based approach, which evaluates the minimally required information to control a desired movement, i.e., control effort. The key idea is to compare the same movement but generated by different actuators, e.g., muscles and torque actuators, and determine which of the two morphologies requires less information to generate the same movement. In this work, for the first time, we apply this measure to numerical simulations of more complex human movements: point-to-point arm movements and walking. These models consider up to 24 control signals rendering the brute force approach of the previous implementation to search for the minimally required information futile. We therefore propose a novel algorithm based on the pattern search approach specifically designed to solve this constraint optimization problem. We apply this algorithm to numerical models, which include Hill-type muscle-tendon actuation as well as ideal torque sources acting directly on the joints. The controller for the point-to-point movements was obtained by deep reinforcement learning for muscle and torque actuators. Walking was controlled by proprioceptive neural feedback in the muscular system and a PD controller in the torque model. Results show that the neuromuscular models consistently require less information to successfully generate the movement than the torque-driven counterparts. These findings were consistent for all investigated controllers in our experiments, implying that this is a system property, not a controller property. The proposed algorithm to determine the control effort is more efficient than other standard optimization techniques and provided as open source.

## 1. Introduction

To generate dynamic movements, biological and technical systems actively process information by sensing their state and deriving control signals. The part of the system that performs this active information processing is typically termed *controller*. A controller has to deal with the dynamics characteristics of the controlled system, e.g., the neuronal delays, and the muscular elasticities and nonlinearities in biological systems or the ideally linear torque characteristics in technical systems. While—from a classical engineering point of view—muscular elasticities and nonlinearities complicate the implementation of an adequate controller, several studies show that they are beneficial for the generation of movements in terms of robustness against perturbations (van Soest and Bobbert, 1993; Gerritsen et al., 1998; Wagner and Blickhan, 1999, 2003; Eriten and Dankowicz, 2009; van der Krogt et al., 2009; Haeufle et al., 2010, 2012; John et al., 2013). Examinations of the control of point-to-point movements in the human arm (Pinter et al., 2012; Kambara et al., 2013; Bayer et al., 2017; Stollenmaier et al., 2020; Wochner et al., 2020) as well as in a frog's leg (Giszter et al., 1993), suggest that the neuronal system explicitly relies on the visco-elastic characteristics of the muscles to stabilize a specific posture or to generate smooth dynamic trajectories from jerky control signals.

When discussing the potential contribution of morphology to control, researchers use conjectures like “reduce the control effort” (Blickhan et al., 2007) or “simplify control” (Full and Koditschek, 1999; Holmes et al., 2006) to suggest that less information has to be processed by the biological controller, i.e., the nervous system, during the movement due to the specific morphology. This part is then performed by the morphology, in the sense of “morphological computation” (Paul, 2006; Zahedi and Ay, 2013; Ghazi-Zahedi et al., 2016). A quantitative analysis of the information processing benefit that is gained by these characteristics of the biological system in direct comparison to (technical) systems with different characteristics is possible. For this purpose, we have proposed to measure the minimally required information to generate a movement, i.e., the *control effort* (Haeufle et al., 2014b). Applied to a simplified model of human hopping—with only one actuator and one mechanical degree of freedom—this approach showed that the muscle properties allow reducing the control effort almost by a factor of 20 in comparison to an ideal torque generator model-driven by a PD controller (Haeufle et al., 2014b). This and the existing evidence for muscular benefits in control suggests that relief of effort for the nervous control system may be engraved into muscle design, and may, in other words, have been one of several basic design criteria during the evolution of biological muscle. We, therefore, hypothesize that control effort is relevant in different and more complex movements.

To study this, we here extend the quantification of control effort to more complex movements as, e.g., human point-to-point arm movements and human walking, which is the first novelty of this paper. To determine control effort in complex musculo-skeletal or robotic models with many control signals, we propose a new algorithm (provided online), which is the second novelty of this paper. We applied this algorithm to two existing musculo-skeletal models: one for arm movements (Driess et al., 2018; Stollenmaier et al., 2020), and one for planar walking (Geyer and Herr, 2010). For each model, a “robotic” version equipped with ideal torque generators was deployed (in analogy to “MOM” in van Soest and Bobbert, 1993). To obtain the controller for point-to-point arm movements, we considered deep reinforcement learning methods. Walking was controlled with proprioceptive neural feedback as well as a PD controller.

## 2. A Summary of the Approach to Quantify Control Effort

The measure of *control effort* previously introduced (Haeufle et al., 2014b) quantifies the minimal information required to generate a specific movement. The basis for this is the quantification of the information of the control signals—i.e., sensor signals and actuator command signals—based on Shannon's information entropy (Shannon and Weaver, 1949). In a nutshell, the idea is to change the resolution of discretized control signals to reduce their information content. If the discretization is too coarse, the movement breaks down. The coarsest resolution where the movement still works represents the minimal information and is termed *control effort*. In the following, we will briefly summarize the concept.

We start with defining parameters for the discretization of the control signals: Each control signal *u*_*i*_(*t*) (with *i* ∈ {1, …, *N*_*u*_} and *N*_*u*_ the number of control signals) is discretized. Discretization limits the number of possible sensor measurement values to *n*_*i*_ (amplitude resolution) and the number of repeated measurements during the movement to *m*_*i*_ (time resolution). Both are positive natural numbers *n*_*i*_ ∈ ℕ_1_ and *m*_*i*_ ∈ ℕ_1_ (excluding 0). Each pair (*n*_*i*_, *m*_*i*_) represents the overall resolution of a specific signal *u*_*i*_. The vector

(1) $$\begin{array}{r} \text{r}=\left(n_{1},m_{1},n_{2},m_{2},\ldots ,n_{i},m_{i},\ldots ,n_{N_{u}},m_{N_{u}}\right)\in R \end{array}$$

is the vector containing all amplitude and time resolution parameters. It has 2*N*_*u*_ elements. The set of possible parameter vectors is $R:={\mathbb{N}}_{1}^{2N_{u}}$, the set of all possible vectors of length 2*N*_*u*_ with positive natural numbers.

The information of these control signals can then be calculated by (see also Appendix A):

(2) $$\begin{array}{r} I\left(\text{r}\right)=\sum_{i=1}^{N_{u}}m_{i}\log_{2}n_{i}. \end{array}$$

with $I:{\mathbb{N}}_{1}^{2N_{u}}\to \mathbb{R}$. This is a simple monotonic function, which depends on the resolution parameter vector. By reducing the values in **r**, i.e., lowering the resolution, the information is reduced.

By reducing the information in the control signal, the movement performance will deteriorate and eventually break down. As an example: if the sensor resolution on the elbow joint position is reduced, the deviation from the target position will eventually increase. To quantify this, we define a performance function $P:{\mathbb{N}}_{1}^{2N_{u}}\to \mathbb{R}$. This performance function is movement specific and will be specified later (see sections 4.1.2, 4.2.2).

Finding the minimally required information, i.e., the *control effort*, is, thus, a constrained optimization problem of the form

(3) $$\begin{array}{cc} \underset{\text{r}\in R}{\min}I\left(\text{r}\right)\\ \text{subject to} & P\left(\text{r}\right)\leq 0\; \end{array}$$

The cost function *I*(**r**) is cheap to evaluate and straight-forward to optimize. Evaluating the constraint *P*(**r**), however, requires the simulation of a movement which is computationally expensive.

## 3. New Algorithm to Quantify Control Effort

In the publication, where we proposed the approach to quantify control effort, we searched for the minimal information by brute force (Haeufle et al., 2014b). This was possible due to the small number of control signals (*N*_*u*_ ≤ 3). For more complex movements with many signals (as investigated here) such an approach needs to be replaced by a systematic optimization. As stated above, it is a constrained optimization problem with a very simple cost function, but a computationally expensive Boolean constraint (movement succeeded or failed), which is additionally stochastic in the presence of motor noise in the investigated arm movements. This prohibits the calculation of a derivative, even by numerical methods. Therefore, all constrained optimization techniques that rely on a gradient of the constraint are not applicable. As the cost function is computationally cheap, it should always be evaluated first and it should be avoided to calculate the constraint function for parameter sets for which it is already clear that the cost is larger than for the currently best parameter set. This makes it difficult to apply algorithms that rely on surrogate functions (e.g., SGHO), as the objective would become very unsteady, also due to the Boolean constraint (a failed movement would be interpreted as a very high cost). Direct search methods seem therefore appropriate (e.g., dual annealing, differential evolution, pattern search). Furthermore, we know two important aspects of our optimization problem: our cost function is monotonically decreasing and if the resolution becomes to coarse, the movement will break down. Thus, we expect a clear border above which the constraint is fulfilled and below it is not. With this knowledge, we can specifically tailor the optimization to minimize the costly calculation of the constraint function. We, therefore, developed a direct search approach that is specifically designed for our optimization problem (Equation 3). The algorithm is based on the pattern search concept, a class of derivative-free direct search algorithms (Todorov and Jordan, 2003; Lewis et al., 2000; Rios and Sahinidis, 2013). In the following, we will describe the concept of the algorithm. Its algorithmic details are given in the Appendix B and the algorithm can be found online at https://github.com/daniel-haeufle/Control_Effort_Optim_Algorithm.

### 3.1. Outline of the Algorithm

In every iteration of the algorithm, a new set of parameters r∈R is selected (polled), evaluated, and the results are compared to the previous best solution (Algorithm 1, Appendix B.1). The key step of the optimization algorithm is the selection (polling) of new parameter sets **r**.

The initial guess of the parameter set **r**_init_ has to be with high values for time and amplitude resolution *n*_*i*_ and *m*_*i*_, almost resembling numerically continuous signals. With the high resolution parameters, **r**_init_ fulfills the constraint function *P*(**r**). Therefore, it becomes the currently best parameter set in the first iteration: **r** = **r**_init_.

Starting from this initial guess, the pattern search algorithm searches for a better solution by exploratory moves (polling) in the parameter space by sampling the function in the vicinity of the currently best parameter set **r**. Polling is performed by iteratively adding a specified set *D* of vectors **d**_*l*_ ∈ *D* (the *pattern*), multiplied by a current mesh size vector $\text{m}\in {\mathbb{R}}^{2N_{u}}$ to the currently best solution as

(4) $$\begin{array}{r} {\text{r}}_{\text{test}}=\text{r}-\text{m}⊙{\text{d}}_{l} \end{array}$$

(where ⊙ represents the element wise multiplication of the two vectors). The mesh size vector basically contains one “scaling factor” for each resolution parameter (entry in **r**). In general, the mesh size vector is reduced (scaled by 0.5) if no better solution is found in the evaluated parameter space and increased (scaled by 2) if a better solution is found. This represents an adaptive search step width (mesh size). For a very helpful overview of the approach of pattern search algorithms, we refer the reader to Torczon (1997).

Our algorithm employs three different polling methods, which differ by the pattern vectors *D*.

#### 3.1.1. Phase 1: Rapid Parallel Reduction of Resolution in All Signals

The first phase is an initial rough sweep where all signals are treated equally. Polling is done by a bisection search method working uniformly on all entries of **r**, i.e., the pattern of this first phase *D*_1_ contains only one vector

(5) $$\begin{array}{r} \bar{\text{d}}=\left(1,1,\ldots ,1\right). \end{array}$$

By adapting the mesh size as described above, this results in a global bisection algorithm acting in parallel on all entries of **r**. The bisection search algorithm is shown in Algorithm 2, Appendix B.2. The benefit of phase 1 is that the performance function needs to be evaluated only a few times to identify a first rough performance limit, even for models with a high number of control signals.

#### 3.1.2. Phase 2: Pattern Search

The more thorough sweep of the second phase is more closely inspired by pattern search algorithms. The pattern *D*_2_ consists of the vectors **d**_*l*_:= *e*_*l*_, where *e*_*l*_ = (0, …, 0, 1, 0, …, 0) is the *l*-th unit vector. It is motivated by the fact that the cost function is monotonically decreasing for each entry. Thus, with this set, the algorithm only polls in the direction of reduced cost *I*(**r**) saving a lot of computational time to less specific search patterns. The vectors **d**_*l*_ represent a linearly independent basis and only modify each variable individually. This is fine for most cases, but may cause the optimization to converge to an undesired local minimum. To reduce this risk, we added a set of vectors $\left\{{\bar{\text{d}}}_{l}\right\}$ in case the previous poll did not reveal any new and better solution. These vectors were constructed such that they had a positive value of 0.5 added to the entry of the previous successful polling direction: $\bar{{\text{d}}_{l}}={\text{d}}_{l}+\left(0,\ldots ,0.5,\ldots ,0\right)$. Let us say the previous successful poll modified the second entry of **r**. Then, the additional polling vectors would look like this:

$$\begin{array}{l} {\bar{\text{d}}}_{1}\;=\left(1,-0.5,0,\ldots ,0\right)\\ {\bar{\text{d}}}_{2}\;=\left(0,0.5,0,\ldots ,0\right)\\ {\bar{\text{d}}}_{l}\;=\left(0,-0.5,\ldots ,1,\ldots ,0\right)\\ {\bar{\text{d}}}_{2N_{u}}=\left(0,-0.5,0,\ldots ,1\right)\;. \end{array}$$

These additional vectors represent linear combinations and allow the optimization algorithm to go “back” in one parameter to get out of a local minimum.

Please note: the mesh size vector **m** is also adapted as in phase 1. The algorithm for phase 2 is shown in Algorithm 3 Appendix B.3.

#### 3.1.3. Phase 3: Check Local Neighborhood and Calculate Error

The third and final phase is used to scan the local neighborhood of **r**_best_ for better solutions and, at the same time, to calculate the error Δ*I*_opt_. We allow for three simultaneously changed entries in **r** as linear combinations of the vectors in the pattern *D*_2_ to find potentially better solutions. For this systematic sweep, the mesh size vector is not adapted anymore. It is simply a vector of ones. This is shown in Algorithm 4, Appendix B.4. In principle, allowing more than three non-zero entries in *d* may further improve the found vector **r**. However, this would come with a high computational cost.

### 3.2. Optimal Result: Control Effort *I*_min_

At the end of the third phase, **r**_opt_ = **r** represents the best parameter vector found by the algorithm. With this, we calculate the control effort, which is the actual information content (Equation 15, Appendix A) of all signals

(6) $$\begin{array}{rc} I_{\min} & =I^{\text{Sh}}\left({\text{r}}_{\text{opt}}\right) \end{array}$$

(7) $$\begin{array}{r} =\overset{N_{u}}{\underset{i=1}{\sum }}\overset{n_{i}}{\underset{j=1}{\sum }}p_{ji}^{\text{opt}}\log_{2}p_{ji}^{\text{opt}}. \end{array}$$

This is the minimally required information content of all control signals to generate the desired movement, i.e., still fulfilling the performance constraint *P*(**r**) = 0. We identify this minimal information as control effort and symbolize it with *I*_min_.

Please note that during the optimization, we assumed equal distribution of the signal values *u*_*ji*_ in the range $u_{i}^{\min}\leq u_{ji}\leq u_{i}^{\max}$ with *j* = 1, …, *n*_*i*_. Therefore, the probabilities were assumed to be *p*_*ji*_ = 1/*n*_*i*_. The cost function of the optimization is based on this assumption and therefore requires no computationally expensive simulation to evaluate the cost function. However, the actual probability *p*_*ji*_ = *p*(*u*_*i*_(*t*) = *u*_*ji*_) that a signal *u*_*i*_(*t*) has the value *u*_*ji*_ at time *t* differs from the original assumption. Therefore, the actual information at the optimal solution differs too. The real probabilities were estimated from the recorded control signals of the optimal walking simulation ($p_{ji}^{\text{opt}}$) using (Equation 7).

### 3.3. Error Estimation

We want to quantify the amount of error that we make by confining the components **r** to integer values. To this end, our search algorithm calculates an error Δ*I*_opt_ specifying the maximum information reduction that can be achieved by reducing a single entry of **r**_opt_ by one. Specifically, we define

(8) $$\begin{array}{r} \Delta I_{\text{opt}}:=\underset{l\in \left\{1,\ldots ,2N_{u}\right\}}{\max}I\left({\text{r}}_{\text{opt}}\right)-I\left({\text{r}}_{\text{opt}}-e_{l}\right). \end{array}$$

For small Δ*I*_opt_, we thus expect the discretization of **r** to only have little effect on the continuous information *I*_min_ ∈ [0, ∞).

## 4. Control Effort in Typical Human Movement Tasks

This study hypothesized that reduced control effort for muscle models over torque actuators found in a simplified hopping model (Haeufle et al., 2014b) is also present in more realistic models of human movements. To test this, we applied the measure described above to biologically plausible models of human point-to-point arm movements and human walking. The models we employed for this study (or very similar ones) have been previously used to study motor control phenomena, where muscle characteristics play a role. Such arm models were used to investigate hypotheses on the control of fast arm movements (Kistemaker et al., 2006), motor learning to compensate for loads during arm movements (Gribble and Ostry, 2000), or the reaction to external forces (Stollenmaier et al., 2020). The walking model was originally used to demonstrate that level walking could be generated by simple reflex control schemes in the spinal cord and does not necessarily require central planning or pattern generators (Geyer and Herr, 2010). It reproduces human muscle activity patterns, joint torques, and kinematics quite well.

**Figure 1:**
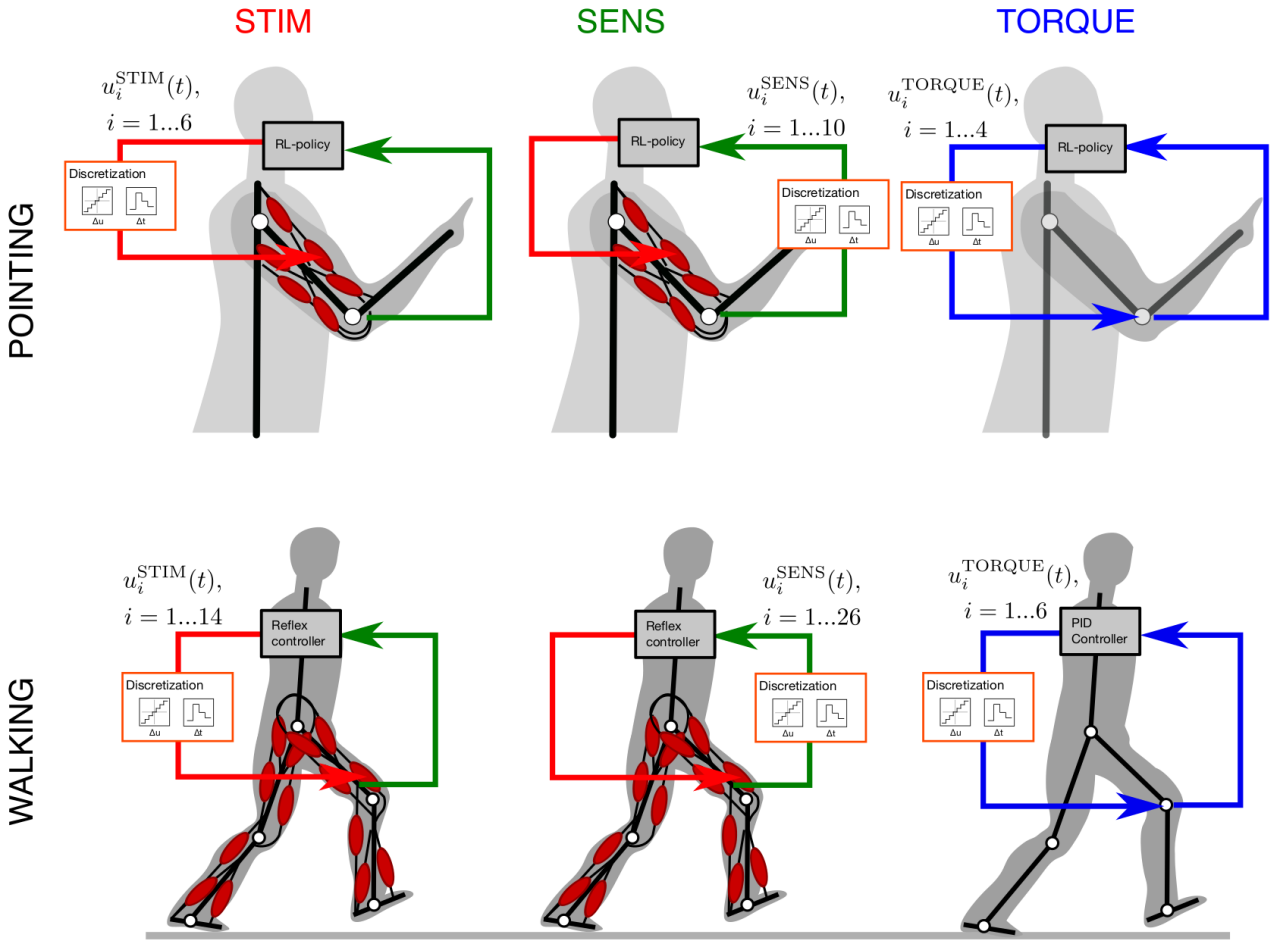
Schematics of the study design: the two movements investigated in this study are goal-directed pointing movements (POINTING) and periodic level walking (WALKING). The pointing movements were simulated with a model consisting of two rigid bodies (upper and lower arm) connected by two hinge joints (based on Stollenmaier et al., 2020). The walking model has seven rigid bodies (two legs with foot, shank, thigh and a single head-arms-trunk segment), all connected by six hinge joints (based on Geyer and Herr, 2010). The muscle-driven models considered nonlinear visco-elastic muscle characteristics and muscular activation dynamics, six muscles in the pointing, 14 in the walking model. The torque-driven models use ideal torque actuators in each hinge joint. The control policy in the pointing models (RL policy) is derived by reinforcement learning. Walking in the muscle-driven model is generated by a reflex-based neural control scheme (Geyer and Herr, 2010) and by a PID controller in the torque-actuator model. To determine *control effort*, the control signals are discretized in amplitude (Δ*u*_*i*_) and time (Δ*t*_*i*_). In the STIM and TORQUE scenario, this discretization is applied to the output of the controller, i.e., the muscular control signals or the torque signals, respectively. In the SENS scenario, the input to the controller is discretized, i.e., the proprioceptive sensor signals.

In both cases, muscle-driven models were the starting point. For comparison, we derived torque-driven models by stripping these models of all muscular dynamics and considering direct torque actuators in the joints. This resulted in a total of six different cases for which we quantified control effort: two different movements with three different scenarios to quantify control effort each. The two movements were goal-directed (pointing) arm movements and level walking. The three different scenarios to quantify control effort were the following (Figure 1)

- STIM: discretization of the motor signals stimulating the muscles (controller output) in the neuromuscular model.
- SENS: discretization of the sensor signals fed back to the controller (its input) in the neuromuscular model.
- TORQUE: discretization of the motor signals (controller output) fed to ideal torque generators.

STIM and SENS represent the biological, neuromuscular system. TORQUE represents the robotic, technical system. In the following, we compare control effort for biological and technical systems in the same movement task.

### 4.1. Movement 1: Pointing

#### 4.1.1. Models

The first movement investigated was a point-to-point arm motion simulated with a 2D arm model (Driess et al., 2018; Stollenmaier et al., 2020). The task is to reach a certain goal position, which also defines the performance criterion *P* and is described in section 4.1.2 below. The arm model consists of two segments representing the upper and lower arm, which are connected by the elbow and shoulder joint to the fixed shoulder (Driess et al., 2018).

We considered two different ways to generate the actuation torques at the joints: First, a muscle-driven arm model using six Hill-type muscle-tendon units—four monoarticular and two biarticular muscles—that produce torques through nonlinear moment arms. The model of the muscle-tendon units considers the nonlinear force-length-velocity characteristics of the muscle fibers, the nonlinear elasticity of the tendon (Haeufle et al., 2014a), and the biochemical processes leading from neuronal stimulation to muscle force (Hatze, 1977). With this, it considers the visco-elastic and low-pass filter properties of muscles, which are considered to be important for stabilizing movements (Gerritsen et al., 1998; Haeufle et al., 2010; Pinter et al., 2012; John et al., 2013). The details and the parameters of the model can be found in Supplementary Material (Data set 1).

As a second model to generate the actuation torques, we simply considered two ideal torque actuators that act directly on the joints of the arm.

In both cases, we used a deep reinforcement learning (RL) algorithm to obtain a controller for reaching a certain goal position. Details about the RL algorithms can be found in Appendix C. We use Deep RL since it bears parallels to biological learning (Neftci and Averbeck, 2019), and the task is simple enough so that we can find good controllers using such a very general learning scheme. The goal of the RL algorithm is to find a policy π which maps an observation (related to the state of the arm model) to an action (the control input), hence a closed loop controller, such that the expected sum of rewards $𝔼\left(\overset{T}{\underset{t=1}{\sum }}r_{t}\right)$ is maximized. A high reward here corresponds to a low deviation from the target position and low applied muscle activations resp. torques. See Appendix C for a proper mathematical definition of these terms.

In our case, the simulation is interrupted every 10 ms in order to get a new control input. We do this for a fixed number *T* ∈ ℕ of iterations. In each of the *T* iterations, the simulation yields a state *s*_*t*_. From the state, we compute an observation *o*_*t*_ = *f*(*s*_*t*_). A given policy π then yields a probability distribution π(*a*_*t*_|*o*_*t*_) from which an action *a*_*t*_ is sampled. This action either corresponds to (normalized) muscle stimulations or to torques. Due to the sampling of the probability distribution, this action has small stochasticity included, similar to motor noise. A fixed reward function *R* is used to compute a reward *r*_*t*_ = *R*(*s*_*t*_, *a*_*t*_). Using the action *a*_*t*_ as a control input, another 10 ms of movement is simulated and the next state *s*_*t*+1_ = *S*(*s*_*t*_, *a*_*t*_) is obtained.

**Figure 2:**
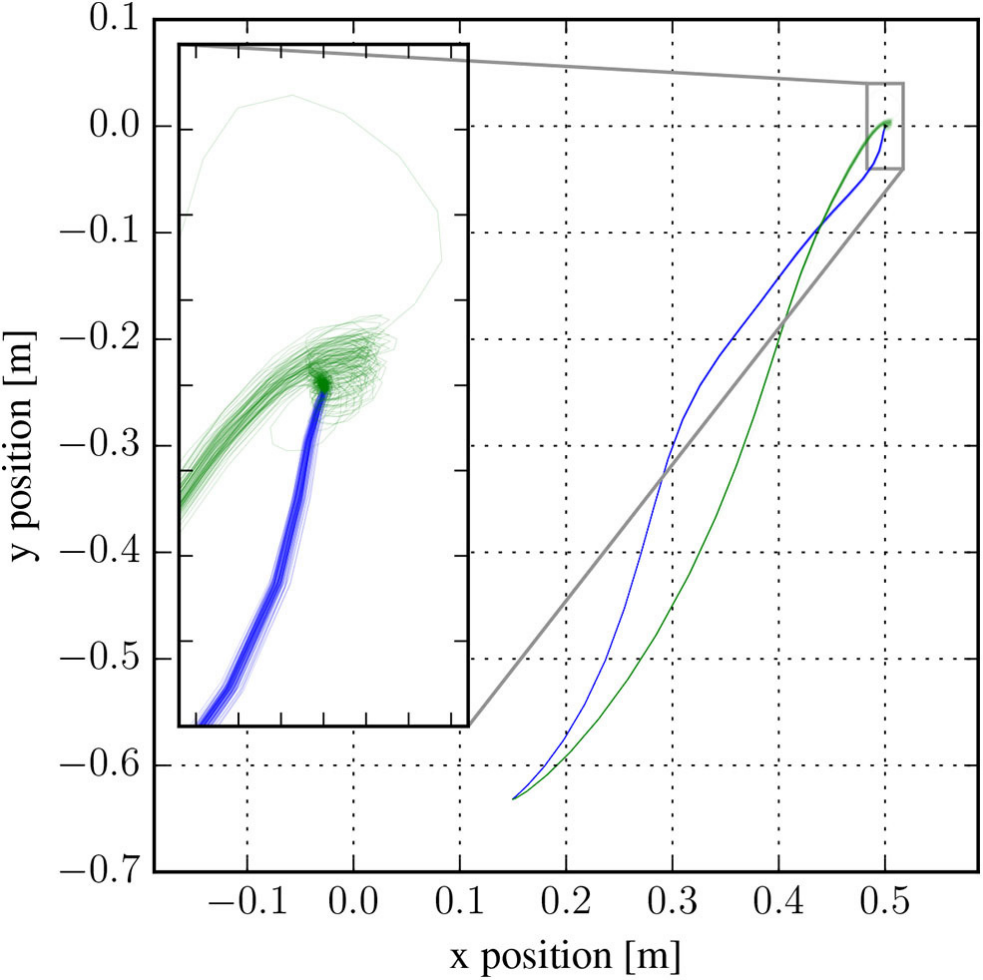
Overlay of 100 sampled trajectories of the end-effector position in the best trained models for the muscle-driven arm (green) and the torque-driven arm (blue). The end-effector moves from its initial position (lower left) to its goal (0.5, 0). Noise in the trajectories arises from noise in the controller (policy) π(*a*_*t*_|*s*_*t*_). The data shown here was generated without delay in the control loop for both models.

Note that the same RL algorithm was used to learn policies for both models (muscle- and torque-driven arm), but the dimensions of the action and observation spaces differ among these models (cf. Appendix C). By construction of this RL algorithm, the distributions π(*a*_*t*_|*o*_*t*_) are Gaussian, i.e., the output of the policy always contains additive Gaussian noise with non-zero variance. More specifically,

$$\begin{array}{ll} \pi \left(a_{t}|o_{t}\right) & =N\left(a_{t}|\text{NN}\left(o_{t}\right),\mathrm{diag}\left(e^{2s_{1}},\ldots ,e^{2s_{d_{a}}}\right)\right)\;, \end{array}$$

i.e., *a*_*t*_ follows a Gaussian distribution with mean given by a learnable neural network applied to *o*_*t*_ and a diagonal covariance matrix with learnable parameters *s*_1_, …, *s*_*d*_*a*__, where *d*_*a*_ is the dimension of *a*_*t*_. While the RL algorithm can adapt the variance of the noise during training, Faisal et al. (2008) suggest that humans can also manage to reduce noise in the nervous system by various complicated mechanisms that cannot easily be modeled or are not yet fully understood. Figure 2 shows that the generated trajectories still contain remaining noise, especially toward the end of the muscle-arm trajectory.

Another difference between the muscle- and torque-driven arm is that we trained and tested the control policy for the muscle-driven model with a delay for sensor signals of 30 ms similar to the electromechanical delay (Mörl et al., 2012; Rockenfeller and Günther, 2016) in human muscles (De Vlugt et al., 2006), i.e., using *o*_*t*_ = *f*(*s*_*t*−3_). We did not consider any control signal delays in our torque-driven model(s) as such delays can be neglected in real-world technical systems that employ torque drives.

#### 4.1.2. Nonlinear Constraint: Movement Performance for Pointing

For pointing movements, we selected as performance criterion *P* the accuracy of pointing to a specific point in space. For each poll, five simulation runs were performed to ensure that the stochasticity of the controller does not affect the result. Accordingly, it was checked as a first part of the criterion *P* whether the arm model's “finger” trajectory ended up in a circle around the desired end goal *x*_goal_ with radius 2.5 cm:

(9) $$\begin{array}{r} {\left(x_{\text{goal}}-x\left(t_{\text{end}}\right)\right)}^{2}+{\left(y_{\text{goal}}-y\left(t_{\text{end}}\right)\right)}^{2}<{\left(2.5\;cm\right)}^{2}. \end{array}$$

Note, that the mean over the five simulation runs was taken for the end position of the trajectories *x*(*t*_end_) and *y*(*t*_end_) to account for the effect of movement variability. The second part of *P* is necessary to ensure that the “finger” not only passes through the target but actually holds this position. Therefore, it was checked whether both angle velocities $\overset{∙}{q_{i}}$ (again averaged) were smaller than a certain threshold:

(10) $$\begin{array}{r} \overset{∙}{q_{i}}<0.15\;\text{rad}/\text{s},\;\text{with }i=1,2. \end{array}$$

Only if both criteria were fulfilled, the poll was considered successful, which gives a conditional expression for the performance criterion as follows:

$$\begin{array}{l} P\left(\text{r}\right)=\{\begin{array}{cc} 0, & \text{if Equation}\left(9\right)\text{and Equation}\left(10\right)\text{are true}\\ 1, & \text{otherwise.} \end{array} \end{array}$$

### 4.2. Movement 2: Walking

#### 4.2.1. Models

The second movement investigated in this study was human walking as “defined” by the performance criterion *P* described in section 4.2.2 below. For this, we resorted to an existing neuromuscular model (Geyer and Herr, 2010). It is a multi-body model with seven segments and hinge-joints in the ankle, knee, and hip. It is actuated by 14 Hill-type muscle-tendon complex models. The muscular control is based on neuronal reflex pathways processing in total 24 proprioceptive signals with biologically realistic neuronal delays. Such a control concept is inspired by the presence of mono-synaptic reflex pathways in the spinal cord, which could explain the low-level implementation of the rhythmic pattern generation of level walking (see Geyer and Herr, 2010, for more details). In forward-dynamic simulations, this model predicts robust walking patterns with strikingly realistic kinematics, ground reaction forces, and muscular activities.

Like for the arm model, we derived a technical model, which, in our case, is a torque-driven model without muscle-tendon characteristics and without neuronal control. This model had the same anthropometrics as the neuromuscular walking model. However, the joint torques for each of the six joints were generated based on PD controllers enforcing the joint kinematics $\varphi_{i}^{\text{ref}}$ recorded from a reference simulation using the neuromuscular model (Geyer and Herr, 2010):

$$\begin{array}{l} u_{i}^{\text{TORQUE}}=k_{P}\left(\varphi_{i}-\varphi_{i}^{\text{ref}}\right)+k_{D}\left({\overset{∙}{\varphi }}_{i}-{\overset{∙}{\varphi }}_{i}^{\text{ref}}\right), \end{array}$$

with *i* = 1…6 for the six joints (2x ankle, 2x knee, 2x hip). This represents a typical low-level control implementation in classical robotics. We here ignore all potential higher-level planning contributions and replace them with the recorded kinematics as the desired trajectory. This is, therefore, equivalent to the level of investigation in the reflex-driven neuromuscular model.

The joint torque was limited to 1.5 times the maximum active values generated by the muscles in the neuromuscular reference simulation. Two sets of feedback gain parameters *k*_*P*_ and *k*_*D*_, one for stance and one for swing phase were determined in simulations with very fine discretization (*n* = 10^15^ and *m* = 10^15^) by a pattern search algorithm (Matlab (R) global optimization toolbox, with random initial conditions). We will show the results for the two best control parameter sets (CP2 and CP10).

The multi-body dynamics of both models are implemented in SimMechanics 1st generation within Matlab(R), SimulinkTM version 2016a. The differential equations are solved with a variable step solver (ode23s stiff/Mod. Rosenbrock) with relative and absolute tolerance of 10^−3^ and 10^−4^, respectively. After an initial phase of approximately 5 s, the model's walking pattern is fairly repetitive. Therefore, all evaluations were done on the interval *t* ∈ [5 s, 10 s].

#### 4.2.2. Nonlinear Constraint: Movement Performance for Walking

The nonlinear constraint for the walking model was a combination of a criterion for a desired walking speed and a second one for “not falling”: From the continuous walking simulation, we can estimate the typical walking speed of the head-arms-trunk (HAT) segment with linear regression to ${ẋ}_{\text{HAT},\text{cont}.}=1.33\;ms^{-1}$. The first criterion for the performance limit is for the x-coordinate of the HAT segment *x*_HAT_ to stay within 6% to this walking speed:

(11) $$\begin{array}{r} \frac{\left|{ẋ}_{\text{HAT},\text{cont}.}-{ẋ}_{\text{HAT}}\left(t\right)\right|}{{ẋ}_{\text{HAT},\text{cont}.}\left(t\right)}<0.06 \end{array}$$

The second criterion—not falling—is simply described by the vertical position of the HAT segment *y*_HAT_. Simulation tests showed that if the condition

(12) $$\begin{array}{r} y_{\text{HAT}}\left(t\right)>1.24\;m \end{array}$$

is violated, the model is falling and not walking anymore.

If at least one of these criteria is violated, the simulation stops (at *t*_stop_) and the performance is the time difference to the desired simulation time

(13) $$\begin{array}{r} P\left(\text{r}\right)=T-t_{\text{stop}}. \end{array}$$

The optimization constraint

(14) $$\begin{array}{r} P\left(\text{r}\right)=0 \end{array}$$

thus only allows for parameter sets which generate walking patterns not violating the above two conditions during the entire simulation time *T* = 10 s.

### 4.3. Discretization of Signals to Determine Control Effort

In principle, it is not clear which signals need to be discretized to determine control effort. At first sight, all output signals of the controller which directly control the actuators—the muscle stimulation or the joint torque signals—seem the obvious choice. However, also the sensor signals provide important information to the system, so it could also be argued that all input signals to the controller need to be discretized (Haeufle et al., 2014b). Here, we tested three scenarios (Figure 1).

#### 4.3.1. Discretize Muscle Stimulations (STIM)

In the first scenario, we discretized the muscle stimulations both in time and amplitude for all muscles with the algorithm given above. These discretized muscle stimulations $u_{i}^{\text{STIM}}$ are then used as an input signal to each muscle. Because the muscles can be activated between 0 and 100%, we set $u_{i}^{\text{STIM},\min}=0$ and $u_{i}^{\text{STIM},\max}=1$ respectively, as well as the duration of the movement *T*^POINTING^ = 1 s for the pointing movements and *T*^WALKING^ = 5 s. The time and amplitude resolution parameters *m*_*i*_ and *n*_*i*_ of each of the stimulation signals $u_{i}^{\text{STIM}}$ were then varied with the algorithm described above.

#### 4.3.2. Discretize Proprioceptive Sensor Signals (SENS)

In the second scenario, all proprioceptive sensor signals $u_{i}^{\text{SENS}}\left(t\right)$ are discretized in the neuromuscular models (Figure 1). Here the minimum $u_{i}^{\min}$ and maximum $u_{i}^{\max}$ signal values were determined from a not discretized reference simulation. As above, the duration was *T*^POINTING^ = 1 s and *T*^WALKING^ = 5 s and the signal resolution parameters were optimized for minimal information with the algorithm above.

#### 4.3.3. Discretize Torque Actuations (TORQUE)

In the third scenario, we discretized the control signals for the torque-driven system $u_{i}^{\text{TORQUE}}\left(t\right)$ (Figure 1). For the POINTING movement, we set $u_{i}^{\min}=-20\;Nm$ and $u_{i}^{\max}=20\;Nm$ to approximately match the capacities of the muscles in the arm model. For the WALKING movement, the minimum $u_{i}^{\min}$ and maximum $u_{i}^{\max}$ signal values were determined from a not discretized reference simulation and the durations were again *T*^POINTING^ = 1 s and *T*^WALKING^ = 5 s. The signal resolution parameters were optimized for minimal information with the algorithm above.

## 5. Results

**Figure 3:**
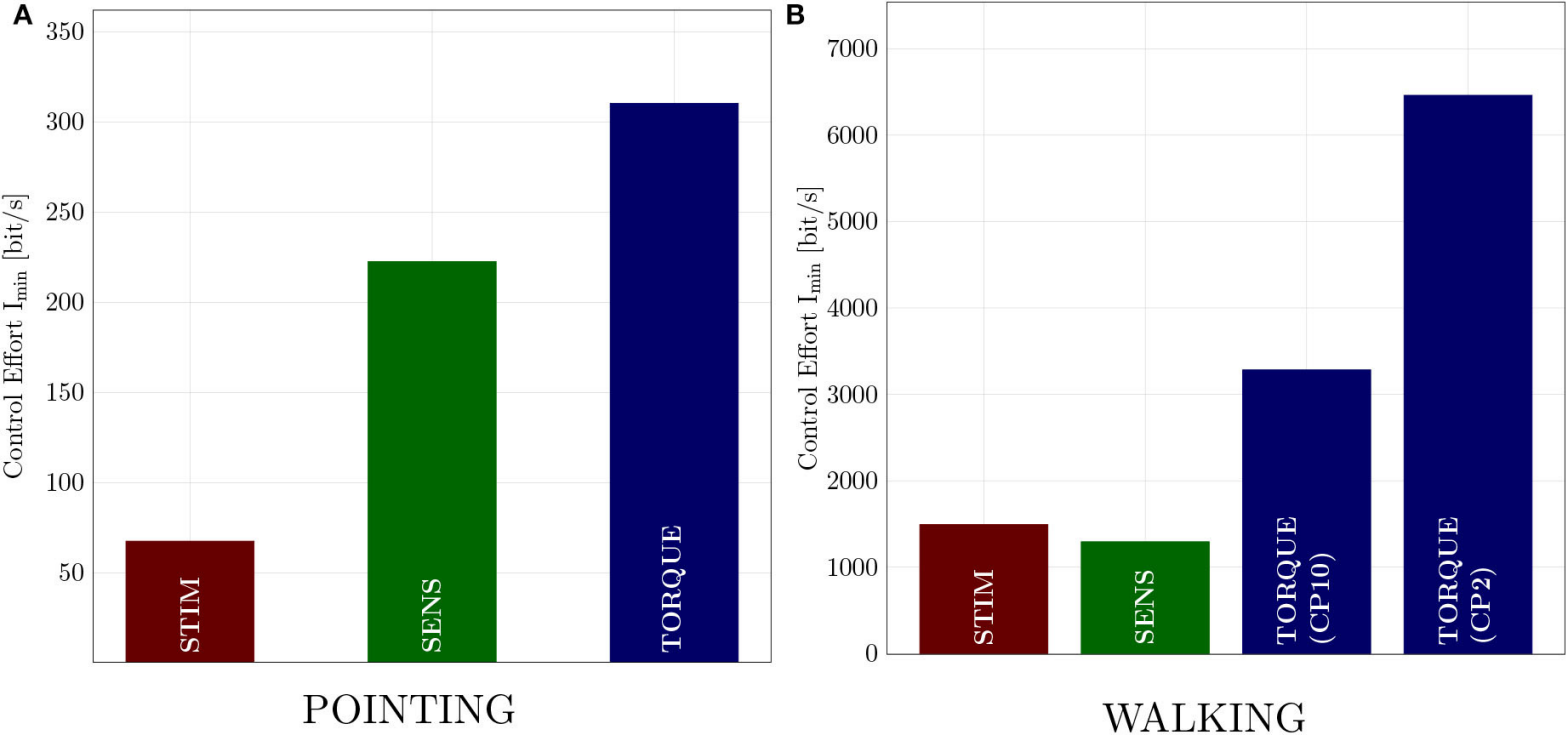
Control effort of **(A)** POINTING movements and **(B)** WALKING. In general, the control effort of walking is higher than the control effort of pointing movements. For both movements, the two neuromuscular models STIM and SENS require less information to generate the motion than the torque-driven model.

The control effort, i.e., the minimally required information *I*_min_ to generate pointing and walking movements, is lower in the neuromuscular models STIM and SENS as compared to the TORQUE model (Figures 3A,B).

In the pointing movements, the control effort is lowest for the STIM scenario ($I_{\min}^{\text{STIM}}=67.5\;bit/s$), where the information is reduced in the output of the controller. The control policy derived by reinforcement learning (RL), however, does not allow to reduce the information as much on the input side (SENS) as on the output side (STIM), resulting in almost three-fold higher control effort ($I_{\min}^{\text{SENS}}=222.5\;bit/s$). The torque model requires the most information, resulting in an almost four-fold higher control effort ($I_{\min}^{\text{TORQUE}}=310.3\;bit/s$).

In the walking model, the control effort for the STIM and SENS scenarios are quite similar and both about half of the best TORQUE model (Figure 3B and Table 1). The second best (CP2) PID controller parameters require double the amount of information than the best parameters (CP10).

**Table 1:** Control effort of walking as determined with the adapted pattern search algorithm at the different stages of the optimization.

**Model**	**Number of control signals *N*_*u*_**	**Initial I *I*_0_[*kbit*/*s*]**	**I stage 1 *I*_1_[*kbit*/*s*]**	**I stage 2 *I*_2_[*kbit*/*s*]**	**Control effort *I*_min_[*kbit*/*s*]**	**Optim. error Δ*I*_opt_[*kbit*/*s*]**
STIM	14	688	3.48 #16	2.68 #611	1.49 #21,715	0.0016
SENS	24	1, 229	7.74 #16	1.88 #3,275	1.29 #67,953	0.0063
TORQUE (CP10)	6	295	295 #16	3.54 #527	3.28 #209	0.0002
TORQUE (CP2)	6	295	220 #16	7.84 #590	6.46 #434	0.0060

*Also given are the number of iterations # for each stage of the optimization. [kbit/s] means [10^3^ bit/s]*.

**Figure 4:**
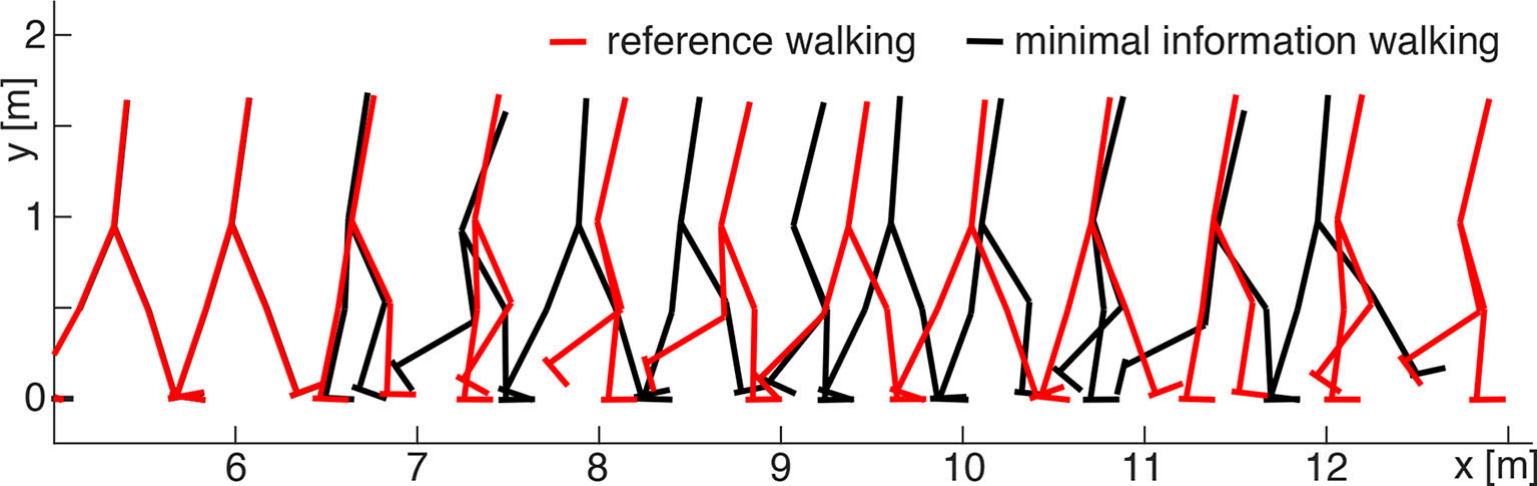
Comparison between walking patterns of the minimal information (SENS model, black) and reference (red) solutions. In the beginning, both solutions overlap until the discretization begins at approximately 6 m walking distance (5 s). Then, the coarse discretization of the minimal information solution affects the walking pattern: the model walks slightly slower than in the reference simulation but still remains within the required performance limit (Equation 11).

**Figure 5:**
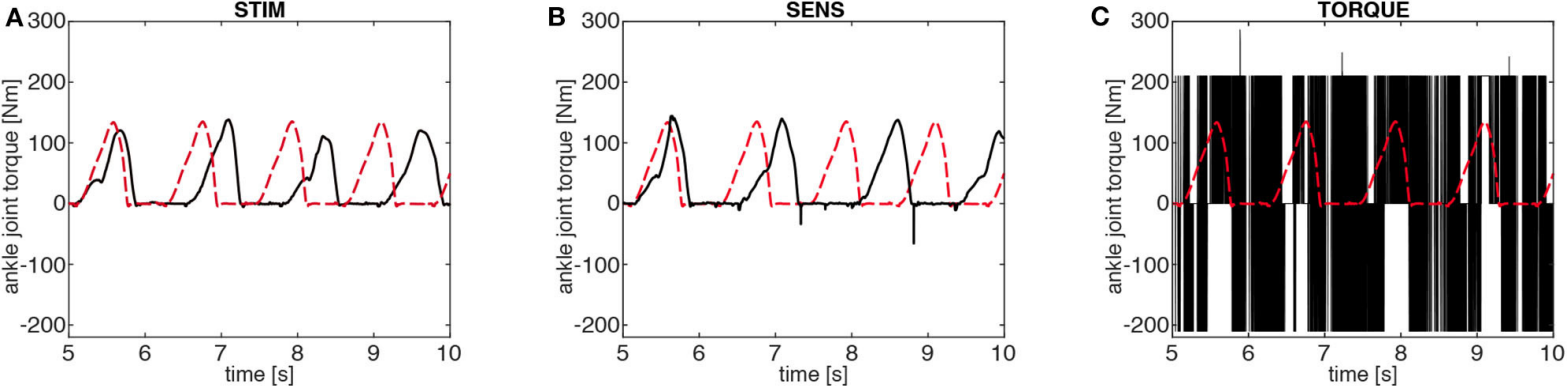
Ankle joint torques of the minimal information solutions. In the STIM **(A)** and SENS **(B)** model, the minimal information solutions (black) involve ankle torques with magnitudes similar to the reference case (red dashed). In the SENS model, the discretization causes over-extensions in the flight phase, which results in short negative torque spikes due to the passive mechanical joint limits of the model. In the TORQUE model **(C)**, the minimal information solution is dominated by a bang-bang pattern between the joint torque limits (±210 Nm). This is a direct result of the coarse discretization of the motor commands $u_{i}^{\text{TORQUE}}$ (see Figure 1).

The discretization—introduced to reduce the information content of the control signals—modifies the walking pattern. In the STIM and SENS scenarios, the parameters **r**_opt_ (minimal information solution) result in slower walking patterns than in the reference solution (Figure 4). In the TORQUE scenario, the parameters **r**_opt_ result in strong oscillations in the joint torques (Figure 5).

To demonstrate the reduction in information by changing the resolution parameters *n*_*i*_ and *m*_*i*_ to lower values (more coarse), we give the results for the different stages of the optimization algorithm (Table 1). The algorithm started with an initial guess of $n_{i}=10^{15}$ and $m_{i}=10^{15}$, resulting in high initial information content *I*_0_ of the control signals which is about two orders of magnitude larger than the optimal result. This initial guess is highest for the SENS model as this model discretizes all 24 sensor signals—the highest number of signals investigated in this study. Therefore, *I*_0_ is naturally high. Also, the number of required iterations is high, especially in the third stage, due to the high number of possible linear combinations checked in this stage. However, at the end of the third stage, the control effort *I*_min_ of the SENS model is the lowest.

**Figure 6:**
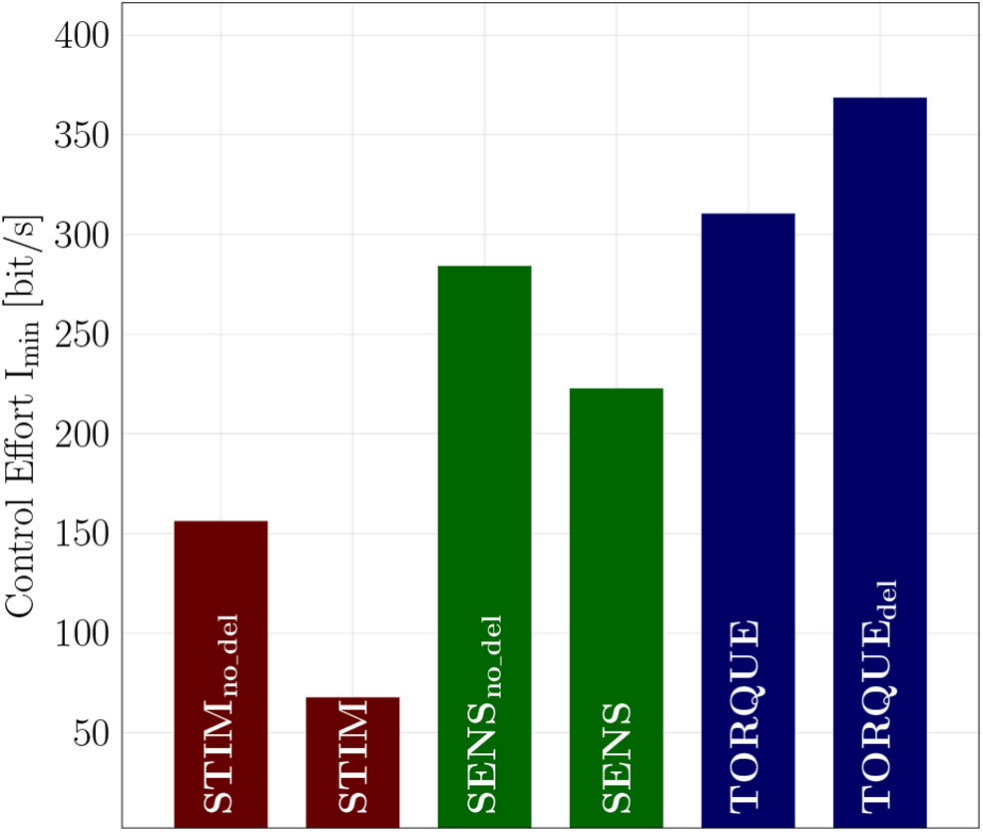
Control effort of the different investigated models, each trained and tested with and without delay for the POINTING movement. The muscle-driven models trained with delay (STIM and SENS) require less information than the corresponding models that were trained and tested without delay (STIM_no_del_ and SENS_no_del_). The TORQUE_del_ model, however, requires more information than the TORQUE model without delay.

In this study (Figure 3A), the model scenarios STIM and SENS were trained and tested with a sensor delay (δ*t* = 30 ms in pointing and δ*t* = 5…20 ms in walking), representing the unavoidable neuronal delay (More et al., 2010) in biology, while the torque model had zero delay representing a modern technical solution. To investigate the influence of sensor delays on control effort in some more depth, we additionally trained and tested control policies for the neuromuscular POINTING movements without delay (unphysiological) in the muscle-driven and with delay (bad engineering) in the torque-driven model. The resulting optimized control effort is shown in Figure 6 in relation to the “correct” models. In the muscle-driven models, the control effort increases in the unphysiological zero-delay scenario, while the torque-driven model benefits from zero-delay.

The estimated error of the control effort in walking Δ*I*_opt_ is small with respect to the difference between the models. The certainty range is an order of magnitude higher than the optimization error. This means that in the last stage of the pattern search, the local neighborhood of the optimal solutions was checked intensively.

Finally, our algorithm is very efficient in finding the control effort. For comparison, we repeated the optimization of the torque model for the pointing movement with other standard optimization algorithms available in Python and found the following: Our algorithm converged in 42 iterations and found a value of 310 bit. In comparison, *dual annealing* stopped at 385 bit after 1000 iterations (the set limit) with 4,067 total function evaluations. *Differential evolution* stopped at 472 bit after 1,000 iterations (the set limit) with 30,033 total function evaluations. *SHGO* did not converge.

## 6. Discussion

Control effort is reduced in muscle-driven systems compared to torque-driven systems for pointing and walking movements. This supports the general notion that muscle contraction (van Soest and Bobbert, 1993; Haeufle et al., 2010) and activation dynamics (Kistemaker et al., 2005; Rockenfeller and Günther, 2018, app. A) can serve as a low-level zero-delay feedback system (preflexes; Brown et al., 1995) supporting the generation and control of dynamic movements (Ekeberg et al., 2004; Proctor and Holmes, 2010). Here, we provide quantitative evidence for its contribution and the potential reduction in information load. From our point of view, this is interesting, because the two typical movements chosen differ greatly in their characteristics and by the number of muscles needed for their generation. Thus, control effort seems to be a general measure for the contribution of morphology to perform a specific task in biological and robotic motion.

Minimization of information processing may be a design principle for shaping bodies and structures during biological evolution (Niven and Laughlin, 2008) as it certainly comes along with the minimization in metabolic energy consumption of the information processing structures themselves (Niven et al., 2007). However, it is *competing* with other movement criteria. A prominent example would be the performance, as demonstrated here, but probably other optimization criteria as well, such as maneuverability, jerk, stability, robustness, accuracy, or reproducibility. Pushing this surely incomplete list of potentially relevant movement criteria to extremes, minimization of control effort is definitely competing with the soundness of body tissue: damage and failure are even more costly than corrections and compensations in movement execution. However, we see that the minimization of information processing may be crucial in the evolution of morphology, and our approach allows us to quantify it.

Having this said, we would like to emphasize that we do not expect the actual system to process only this minimal amount of information. Especially in biology, there is an abundance of structures (Latash, 2012)—many muscles, sensors, and neurons—which are not considered here. Also, in robotics, one would never control a robot at this limit, as it is just on the verge of instability. However, by applying this minimal information approach systematically to the same movement but different morphologies, the contribution of the latter can be uncovered and quantified.

### 6.1. Influence of Delay on Control Effort

Delay in information processing seems per definition unavoidable (Nishikawa et al., 2007; Shadmehr et al., 2010). In robotic systems with their electric cables, the delay can be very small—usually smaller than the typical time resolution Δ*t*. However, it is a universal characteristic of neuronal information processing in biological systems that the delay is, in general, much larger than the time resolution, and scales with the size of the animal (More et al., 2010). Despite these large delays, animals can perform quite well in uncertain environments. In fact, our approach shows that neglecting this delay in the neuromuscular model increases control effort (Figure 6). On the other hand, engineers who employ widespread electric motors do good in trying to minimize delay (Figure 6).

Neuronal systems have additional possibilities that allow them to compensate drawbacks of delays, which are not considered in our models. They may use open-loop control signals—potentially from an inverse model or a model template (Full and Koditschek, 1999; Holmes et al., 2006)—to drive a movement and only use feedback if a perturbation occurs (Todorov and Jordan, 2003). Furthermore, by predicting sensor states with a forward model (e.g., a template), they may deal with possible instabilities arising from delays (Shadmehr, 2010), at least as long as no external perturbation occurs (Kalveram and Seyfarth, 2009). Despite these neuronal capabilities, the control approach can still rely on the stabilizing response of the visco-elastic muscles to external perturbations (van Soest and Bobbert, 1993; Wagner and Blickhan, 2003; Haeufle et al., 2012; Stollenmaier et al., 2020). Brown et al. (1995) termed these responses “preflexes,” due to their zero time-delay response. There are strong indications that such strain-rate-dependent actuator properties, even more in combination with positive muscle force feedback (Geyer et al., 2003), as well as position-plus-rate characteristics of proprioceptors (McMahon, 1984, p. 154–155) can also provide predictive information that is valuable for movement stabilization. Thus, a delay well-tuned to the controller/control-system interaction may even improve performance (Hedrick and Daniel, 2006; Shadmehr, 2010), and potentially allow to reduce the control effort, as our results indicate.

### 6.2. Information Processing in Walking Machines

The processed information in a digitally controlled walking machine can be estimated with Appendix A, Equation 17. Although the necessary parameters would be easy to determine for the construction engineer, they are usually not published. As one example, we estimated the parameters for a walking pattern reported for the robot MABEL from information given in Park et al. (2011) and Sreenath et al. (2011). MABEL seems to be interesting for comparison, as it is a 2D walking machine that considers elasticities in the drive. Based on the data provided by the papers, we estimate the total information processed in MABEL per second to be *I* = 6.4·10^4^ bit/s. The derivation of this is described in more detail in Appendix D.

This value is large in comparison to the minimal information *I*_min_ predicted by our models but low in comparison to our initial guess *I*_0_. Obviously, the choice of encoder resolution is not made to generate walking with the least amount of information. This is very well not recommended in a technical system, as low resolutions entail the risk of significant oscillations, as seen in our optimized TORQUE results. However, with this comparison, we speculate that our results are in a reasonable range. To evaluate the contribution of morphology to the control and to verify our model calculations, it would yet be quite interesting to apply our algorithm to MABEL while further modifying the characteristics of this machine's actuators.

### 6.3. A Hypothetical Scenario Where an Ideal Torque Generator Would Be Advantageous

Above, we exclusively cited papers indicating and demonstrating the benefit of muscles, and our results fit in this picture. Therefore, it is important to point out that control effort cannot be expected to always be lower in muscle-driven systems. For this, we would like to perform a short though experiment. Imagine a hypothetical task: an arm with a single joint has to generate exactly the same tangential endeffector force at any given joint angle (at rest). An ideal torque generator, would require a single constant input signal. Such a signal, per definition of Equation (2), contains the minimally possible control effort as the resolution parameters could be reduced to *m* = 1 and *n* = 1, and, consequently, *I*_min_ = 0. A muscle-actuated arm, on the other hand, would have to adapt the stimulation to each and every angle as the muscle force depends on its length and therefore also on the joint angle. This is only to highlight that muscles are very well suited for particulary dynamic tasks, but not in general the best actuator for everything.

### 6.4. Other Optimal Control Approaches for Measuring Simplicity

In the present work, we utilize control effort, which has recently been proposed by us (Haeufle et al., 2014b), to quantify the minimal information required to generate a specific movement. This measure is based on the quantification of the information of the control signals, i.e., sensor signals and actuator command signals, based on Shannon's information entropy (Shannon and Weaver, 1949, see section 2). By comparing control effort for different morphologies, it quantifies, to some extent, how “simple” it is to generate a specific movement depending on the morphology of the system.

Brockett (1997) argue to consider simplicity as a way to *synthesize* controllers, which they call Minimum Attention Control (MAC). In order to measure simplicity, they introduce the concept of attention, which quantifies the required rate of change of the control to achieve desired changes in the system state. This can be interpreted as the difficulty to implement a respective controller (Brockett, 1997). For example, a control system which can be controlled by just a constant input would require minimal (no) attention. Thus, the basic idea is to find controllers through an optimal control framework where the objective function trades-off system performance with attention, i.e., simplicity of the controller.

In Della Santina et al. (2017), MAC was found as a beneficial solution for controller design in soft robots. Biomechanical systems such as the arm model used in the present work have properties which enable to reach a desired system state with a constant control input. This has been exploited in Driess et al. (2018), Wochner et al. (2020) and Driess et al. (2019) to learn a controller for such systems efficiently. The controllers of Driess et al. (2018) and Driess et al. (2019), Wochner et al. (2020) are, by design, optimal with respect to attention with zero attention, since the controller produces constant controls for each desired system state.

The measure of attention from Brockett (1997) is, in a way, similar to control effort of the present work, as it is also driven by the idea that a specific design of a control system could be beneficial to achieve a certain system behavior without an overly complex controller. Thus, the process of evolving structures and functioning—simultaneous and codependent control system and controller design—can also be supported by MAC.

However, there is also an important difference between MAC and control effort as considered in the present work. MAC is a paradigm to synthesize controllers by integrating it directly into the cost function of an optimal control framework. In contrast, we use control effort here as a measure to analyze the contribution of the systems dynamics to the control of the movement. Therefore, it measures a system property. Indeed, the controllers that are either learned or hand-tuned in this work at no point have the objective to minimize control effort. Solving the optimal control problem with MAC as an objective is non-trivial, especially for nonlinear systems. In the future it could be investigated whether MAC can be extended to nonlinear biomechanical models and to test whether it allows to find controllers that show a difference in attention between musculoskeletal and torque-driven actuators.

### 6.5. The Optimization Algorithm

Quantifying control effort requires to solve an optimization problem (Equation 3). The algorithm proposed here is novel and specifically designed to efficiently optimize the given problem. The three stages of the algorithm differ in their computational expense, with the first stage being computationally cheap (16 iterations for the walking model), while the other two require more iterations (Table 1). For the few control signals discretized in the TORQUE model, the final search in stage 3 is also computationally cheap. For more control signals, the linear combinations tested in the third stage are computationally expensive. It may be considered to exclude the final stage, as we did for the POINTING movements, since the difference in the results between stage two (*I*_2_) and final result (*I*_min_) in the walking model are not very large, and the general trend can already be seen. In general, this algorithm can easily be applied to any other simulation of movements, and also to robotic systems (which would, however, require safety measures to avoid damage in the low-resolution trials). By providing it as open source, we hope to foster the quantitative evaluation of control effort and a more systematic study of the contribution of morphology to control in biological systems.

## Data Availability Statement

The datasets generated for this study are available on request to the corresponding author.

## Author Contributions

DFBH, MG, and SS: project idea. DFBH, SS, and MG: control effort algorithm. DFBH, IW, DH, and DD: model. DFBH and IW: data. All authors: paper.

## Conflict of Interest

The authors declare that the research was conducted in the absence of any commercial or financial relationships that could be construed as a potential conflict of interest.
