# Supplementary material for: Muscles Reduce Neuronal Information Load: Quantification of Control Effort in Biological vs. Robotic Pointing and Walking
Source: Front Robot AI. 2020 Jun 24;7:77. doi: 10.3389/frobt.2020.00077 (PMC7805995; doi:10.3389/frobt.2020.00077)
Supplement: Supplementary file 2 [file Data_Sheet_2.pdf]

Appendix to: Muscles reduce neuronal information load: quantification of control effort in biological vs robotic pointing and walking

Daniel F. B. Haeufle Isabell Wochner David Holzmüller,
Danny Driess, Michael Günther, Syn Schmitt

June 3, 2020

The original publication can be found at
https://doi.org/10.3389/frobt.2020.00077.

A. Information of a control signal

In this section, we give the reasoning for Eq. 2. This is a summary of the derivation shown in (Haeufle et al., 2014).

The average amount of information gained in a measurement, e.g., from a sensor or other control signal, can be calculated based on SHANNON's information entropy (Shannon and Weaver, 1949):

$$I^{\text{Sh}} = -K \sum_{j=1}^{n} p_j \log_2 p_j \quad , \tag{15}$$

where $p_j = p(u = u_j)$ is the probability of the specific signal value $u = u_j$, n is the number of possible signal values, and $\sum p_j = 1$. The constant $K = 1\,\text{bit}$ defines the unit of information. This concept is the basis for our measure of control effort.

In our approach, we assume discretized signals measured at constant time intervals as typical for digital technical systems and easily implementable in computer simulations (Haeufle et al., 2014). Typically, a control system processes many signals. Each signal $u_i(t)$ is discretized in amplitude by intervals of the size Δu_i and in time to Δt_i. Here, $i = 1, \ldots, N_u$ applies, with N_u being the number of signals contributing to the control. If the minimum and maximum signal values $u_i^{\min} = \min(u_i(t))$ and $u_i^{\max} = \max(u_i(t))$ are known, it is possible to determine the number of possible signal values

$$n_i = 1 + \frac{u_i^{\max} - u_i^{\min}}{\Delta u_i} \quad .$$

If the total duration of the movement T is known, the number of repeated measurements is

$$m_i = 1 + \frac{T}{\Delta t_i} \quad .$$

With the assumption of equal distribution of the measurement values (Haeufle et al., 2014), this allows the following simplified estimate for a signal's information (Eq. 15)

$$\begin{aligned} I &= \sum_{i=1}^{N_u} m_i \log_2 n_i && (16) \\ &= \sum_{i=1}^{N_u} \left(1 + \frac{T}{\Delta t_i}\right) \log_2 \left(1 + \frac{u_i^{\max} - u_i^{\min}}{\Delta u_i}\right) \quad . && (17) \end{aligned}$$

This estimate shows that the information in the signals depends on the time and amplitude resolutions. A coarser discretization, meaning smaller m_i and n_i in Eq. 2, or larger Δt_i and Δu_i in Eq. 17, respectively, reduces the information content of a signal. To minimize the information content of the signals, we proposed to optimize the resolution parameters n_i and m_i for minimal information, and associate this minimal information with control effort.

B. Optimization algorithm

The optimization problem (Eq. 3) was solved with a direct search method similar to a pattern search algorithm. The concept of the algorithm was described above (Sec. 3). We here give some additional details on the algorithmic implementation. The full algorithm is available online at https://github.com/daniel-haeufle/Control_Effort_Optim_Algorithm.

Please note that in our Matlab® implementation, the resolution parameters can only be natural numbers $\mathbf{r} \in \mathcal{R} = \mathbb{N}_1^{2N_u}$, however, in principle, they could also be $\mathbf{r} \in \mathbb{R}_{\geq 1}^{2N_u}$.[1] The implementation with natural numbers results in a limit of the of the error ΔI_{opt} which cannot be further reduced. As this error was always smaller than the differences in I_{opt} in our test cases, we kept this approach for easier interpretation of the result.

B.1. Helper functions

In every following iteration a new set of parameters $\mathbf{r} \in \mathbb{R}^{2N_u}$ is selected (polled), rounded up as $\lceil \mathbf{r} \rceil \in \mathcal{R}$, evaluated, and the results are compared to the previous best solution:

B.2. Algorithm of phase 1: rapid parallel reduction of resolution in all signals

In this phase, polling is done by a bisection search method working uniformly on all entries of $\mathbf{r}$. For this purpose, we introduce the vector $\mathbf{m} \in \mathbb{R}^{2N_u}$ which we call mesh-

[1] This would correspond to unequal spacing of the quantized points inside the intervals.

Algorithm 1 Helper functions

function IMPROVES($\mathbf{r}_{\text{test}}, \mathbf{r}$) ▷ Tests whether $\lceil \mathbf{r}_{\text{test}} \rceil$ is feasible and better than $\lceil \mathbf{r} \rceil$
    return $I(\lceil \mathbf{r}_{\text{test}} \rceil) \leq I(\lceil \mathbf{r} \rceil)$ **and** $P(\lceil \mathbf{r}_{\text{test}} \rceil) = 0$
end function
function CLIP($\mathbf{r}$) ▷ Clips $\mathbf{r}$ to be between $\mathbf{1}$ and $\mathbf{r}_{\text{init}}$
    return $\max(\mathbf{1}, \min(\mathbf{r}_{\text{init}}, \mathbf{r}))$
end function

size. The bisection search algorithm is shown in Algorithm 2.

Algorithm 2 Phase 1 of the optimization algorithm.

$\mathbf{m} \leftarrow \mathbf{r}_{\text{init}} - \mathbf{1}$
$\mathbf{r} \leftarrow \mathbf{r}_{\text{init}}$
$\mathbf{r}_{\text{best}} \leftarrow \mathbf{r}_{\text{init}}$
while $\max(\mathbf{m}) > 1$ **do**
    $\mathbf{m} \leftarrow \mathbf{m}/2$
    if $P(\lceil \mathbf{r} \rceil) = 0$ **then**
        $\mathbf{r} \leftarrow \mathbf{r} - \mathbf{m}$
    else
        $\mathbf{r} \leftarrow \mathbf{r} + \mathbf{m}$
    end if
end while

The following example is meant to visualize the polling procedure, which depends on the outcome of the constraint evaluation:

k	n1	m1	n2	m2	n3	m3	constraint
init	16	16	16	16	16	16	yes
2	8	8	8	8	8	8	no
3	12	12	12	12	12	12	yes
4	10	10	10	10	10	10	no
5	11	11	11	11	11	11	yes

B.3. Algorithm of phase 2: pattern search

The second phase of the optimization algorithm is an adapted pattern search algorithm:

The following example is intended to visualize the polling of the second phase. It continues from the final result of the phase 1 example:

k	n1	m1	n2	m2	n3	m3	constraint
r	11	11	11	11	11	11	yes current r

Algorithm 3 Phase 2 of the optimization algorithm.

$\mathbf{m} \leftarrow \mathbf{r}/4$
$D \leftarrow [e_1, \ldots, e_{2N_u}]$ ▷ Represents descent directions
$k \leftarrow 1$ ▷ Represents the last successful descent direction
while $\max(\mathbf{m}) > 1/2$ **do**
  success $\leftarrow$ false
  for $l = 1, \ldots, \mathrm{len}(D)$ **do**
    $\mathbf{r}_{\text{test}} \leftarrow \textsc{Clip}(\mathbf{r} - \mathbf{m} \odot D[l])$ ▷ $\odot$ is the element-wise product
    if $\textsc{Improves}(\mathbf{r}_{\text{test}}, \mathbf{r})$ **then**
      success $\leftarrow$ true
      $\mathbf{r} \leftarrow \mathbf{r}_{\text{test}}$
      if $l \leq 2N_u$ **then**
        $k \leftarrow l$
      end if
      break for loop
    end if
  end for
  if success **then**
    $\mathbf{m} \leftarrow 2\mathbf{m}$
    $D \leftarrow [e_1, \ldots, e_{2N_u}]$
  else
    $\mathbf{m} \leftarrow \mathbf{m}/2$
    $D \leftarrow [e_1, \ldots, e_{2N_u}, e_1 - e_k/2, \ldots, e_{k-1} - e_k/2, e_k/2, e_{k+1} - e_k/2, \ldots, e_{2N_u} - e_k/2]$
  end if
end while

```
6    |  7 11 11 11 11 11 |no   r - m*d_1
7    | 11  7 11 11 11 11 |no   r - m*d_2
8    | 11 11  7 11 11 11 |no   r - m*d_3
...
11   | 11 11 11 11 11  7 |no   m*=0.5
12   |  9 11 11 11 11 11 |no   r - m*d_1
13   | 11  9 11 11 11 11 |yes  m*=2 new r
14   |  7  9 11 11 11 11 |no   r - m*d_1
15   | 11  5 11 11 11 11 |no   r - m*d_2
14   | 11  9  7 11 11 11 |no   r - m*d_3
...
96   | 10  9  7  2  5 11 |yes  current r
```

B.4. Algorithm of phase Phase 3: check local neighborhood

The final step is used to scan the local neighborhood of $\mathbf{r}_{\text{best}}$ for better solutions and, at the same time, to calculate the error ΔI_{opt} and the certainty interval $\Delta I_{\text{certainty}}$. This is

shown in Algorithm 4.

Algorithm 4 Phase 3 of the optimization algorithm.

success ← true
while success **do**
  success ← false
  for $k_1 = 1, \ldots, 2N_u$ **do**
    for $k_2 = 1, \ldots, 2N_u$ **do**
      for $k_3 = 1, \ldots, 2N_u$ **do**
        $\mathbf{r}_{\text{test}} \leftarrow \textsc{Clip}(\mathbf{r} + e_{k_1} - e_{k_2} - e_{k_3})$
        if $\textsc{Improves}(\mathbf{r}_{\text{test}}, \mathbf{r})$ **then**
          success ← true
          $\mathbf{r} \leftarrow \mathbf{r}_{\text{test}}$
          break all for loops
        end if
      end for
    end for
  end for
end while

B.5. Additional remarks on the algorithm

The algorithm itself does not ensure that a parameter set is not polled repeatedly. To avoid such computationally expensive recalculations, we implemented a memory into the nonlinear constraint function. It simply checks if the result has been calculated before and saves new results. Another trick to reduce computational expense is to check the performance limit of the constraint function online during the simulation and abort the simulation as soon as the constraint $P(\mathbf{r}) = 0$ cannot be satisfied anymore.

Although we only allow natural numbers as entries of $\mathbf{r}$ for the evaluation of the cost function, we calculate all mesh size multiplications and divisions with double precision floats and always round the values to the next higher integer value.

C. Reinforcement Learning

C.1. Environment

In this section, the simulation setup for the point reaching (POINTING) task is specified in more detail. The goal is to reach the position $x_{\text{goal}} = (0.5, 0)$ with the hand of a simulated 2D arm that has two rotational joints, one at the shoulder and one at the elbow as shown in Fig. 1. Here, the shoulder joint is fixed at the coordinates $(0, 0)$, and the position of the end-effector (i.e. the hand) is uniquely described by the joint angles $q = (\alpha, \beta)$. We compare a simulation of this human arm model that is driven by 6 Hill-type muscles with a kinematically equivalent version actuated with ideal torque sources directly at the joints.

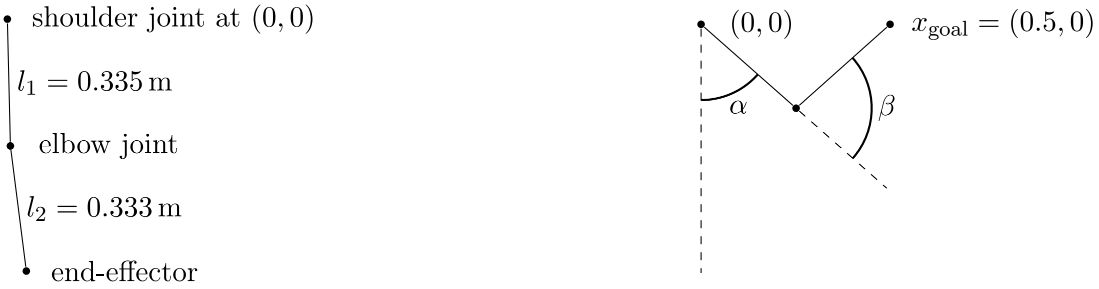

(a) Initial arm position. (b) Desired arm position.

Figure 1: Kinematic model of human arm. Different arm positions, joints and angles.

Muscle-driven human arm model We simulate a human arm with six muscles using our in-house multi-body simulation code `demoa` (see also Supplementary Material based on Stollenmaier et al. (2020)). The arm in initialized in the equilibrium state that the muscle model reaches for the constant input 0.05 on all muscles, which yields angles $q(0) \approx (0.023949, 0.10298)$.

Torque-driven arm model We simulate an arm with the same lengths and masses as in the muscle-driven case, including gravity. We use a custom simulator based on solving the Euler-Lagrange equations with an explicit Euler scheme. Joint limits are treated the same way as in the muscle-based simulation. We use the same initial position and velocity ($q(0) = (0.023949, 0.10298)$ and $\dot{q}(0) = (0, 0)$) as in the muscle-driven case to obtain comparable results

C.2. Reinforcement Learning

To obtain the controller for the arm reaching task, we use deep reinforcement learning (RL). The advantage of this method is that nonlinear (optimal) control policies can be obtained for complex systems by only specifying the goal in terms of a reward function.

In general, an RL algorithm tries to find a policy π which maps an observation o (related to the state of the system) to an action a (the control input) such that the expected sum of rewards $\mathbb{E}\left(\sum_{t=1}^{T} r_t\right)$ is maximized. In our case, π represents the closed-loop controller that generates torques or muscle-stimulations from a measurement of the current state.

More specifically, the simulation is run for a fixed number $T = 100$ of timesteps, where each timestep corresponds to 10 ms, i.e. the motion to reach the target should be completed in less than a second. In each of the T iterations, the simulation yields a state s_t. From this state, an observation $o_t = f(s_t)$ is computed. The policy π then yields a probability distribution $\pi(a_t|o_t)$ from which an action a_t is sampled. A fixed reward function R is used to compute a reward $r_t = R(s_t, a_t)$. Using the action a_t as a control input, the simulation S transitions to the next state $s_{t+1} = S(s_t, a_t)$.

In the following, we describe the action/observation space, the reward function and

the used algorithms in more detail.

Action/control space We use the following domains for the actions a_t:

- The **muscle-driven arm** generally allows inputs between 0 and 1 for all six muscles, which corresponds to normalized muscle stimulations. In order to avoid unrealistically low actions, we require $a_t \in [0.02, 1]^6$.
- The **torque-driven arm** allows for arbitrary torques at both joints. In order to approximately match the capacities of muscles, we restrict the actions (measured in Nm) to $a_t \in [-20, 20]^2$.

Observation space For the torque-driven system, we always provide the full state as observations: $o = s = (q, \dot{q}) \in \mathbb{R}^4$. For the muscle-driven model, we provide $o = (q, \dot{q}, l_{\mathrm{CE}}) \in \mathbb{R}^{10}$. Here, q denotes the joint angles, $\dot{q}$ the angle velocities, and l_{CE} the length of the muscle fiber.

Reward For our purposes, the reward function should balance two objectives:

- Penalizing large distances from the goal: A natural choice is the squared euclidean distance $d^2 = \|x_t - x_{\mathrm{goal}}\|_2^2$ of the hand position to the goal. However, the squared error d^2 only imposes a very small penalty on oscillating motions around the target position. Therefore, we include a logarithmic term as suggested in Lillicrap et al. (2015).
- Penalizing large activation values a_t: Motions with low muscle stimulations or torques should be preferred, respectively. Therefore, the reward contains a term $\lambda_a \|a_t\|_2^2$ with $\lambda_a = 1$ for the human arm and $\lambda_a = 1/200$ for the robot arm. With these weightings λ_a, the term $\lambda_a \|a_t\|_2^2$ is comparably large for trained policies on muscle-driven and torque-driven models.

In summary, the reward is defined as

$$r_t := \frac{1}{10}\left(-d^2 - \log(d^2 + \varepsilon^2) - \lambda_a \|a_t\|_2^2\right),$$

where $\varepsilon := 10^{-4}$ and $d = \|x_t - x_{\mathrm{goal}}\|_2$. The scaling factor $\frac{1}{10}$ is used to avoid issues with NaN values in the ACKTR algorithm.[2]

Delay We optionally provide observations with a delay of 30 ms, i.e. $o_t = f(s_{\max\{0,t-3\}})$ instead of $o_t = f(s_t)$ to investigate typical delays present in biological systems.

[2] https://github.com/openai/baselines/issues/127

Algorithm In order to find a good control policy π, we use ACKTR, a trust-region based policy gradient algorithm that relies on an approximation of the natural gradient (Wu et al., 2017). ACKTR models the policy distribution $\pi(a_t|o_t)$ as a Gaussian distribution. The implementation is from `baselines` (Dhariwal et al., 2017) with adjusted hyperparameters. In particular, best results were obtained with the KL-divergence step size 0.032 for the human arm and 0.064 for the muscle arm. For the policy and value function networks, we use two hidden layers with 64 neurons each. We got worse results using the default implementations of A2C (Mnih et al., 2016), DDPG (Lillicrap et al., 2015), PPO (Schulman et al., 2017) and TRPO (Schulman et al., 2015) from `stable-baselines` (Hill et al., 2018).

D. Control effort in walking machines

Our quantification method can also be applied to real technical systems. As an example, we calculate the processed information for the digitally controlled walking robot MABEL. MABEL seems to be interesting for comparison, as it is a 2D walking machine that considers elasticities in the drive.

The processed information in a digitally controlled walking machine can be estimated with Eq. 17. The required parameters were derived from information given in Park et al. (2011); Sreenath et al. (2011). The authors wrote that the robot's state can be described by the variables $q_e = (q_{\mathrm{LA}}; q_{\mathrm{mLS}}; q_{\mathrm{Bsp}}; q_{\mathrm{Tor}}; p^h_{\mathrm{hip}}; p^v_{\mathrm{hip}})$, which are the leg angle, the position for the leg shape motor, the B-spring pulley position, the torso angle with respect to the vertical, and the horizontal and vertical position of the hip (Sreenath et al., 2011).

The values for u^{min} and u^{max} specifying the signal ranges, and the total duration of the movement T, can be estimated from plots of these variables (Figures 5 and 6 in Sreenath et al., 2011). Unfortunately, no plot or data on the hip positions was given in these publications. The encoders used to determine these signals all have a resolution of 0.1758 deg/count (Park et al., 2011). However, the resolution Δu of some of the signals differs depending on the gearing. Based on Eq. (6) by Park et al. (2011), the resolution for the leg angle is

$$\Delta u_{LA} = \frac{1}{-23.53}\Delta q_{mLA} = 0.00747\,\mathrm{deg}$$

and the gearing of the torso angle is 3:1 resulting in a resolution $\Delta u_{Tor} = 0.05859\,\mathrm{deg}$ (Park et al., 2011). Based on these parameters and considering that two legs are used for walking, the total information can be estimated to be 64 137 bit/s (see Tab. 1). This value is large in comparison to the minimal information I_{min} predicted by our models but low in comparison to our initial guess I_0.

Table 1: Parameters required to estimate the information processed in walking by the robot MABEL derived from Park et al. (2011); Sreenath et al. (2011).

Signal	usage	u^{min}	u^{max}	Δu	T	Δt	I per cycle
q_{LA}	2 times	171 deg	204 deg	0.00747 deg	0.72 s	0.001 s	8716 bit
q_{mLS}	2 times	111 deg	530 deg	0.1758 deg	0.72 s	0.001 s	8008 bit
q_{Bsp}	2 times	0 deg	14.5 deg	0.1758 deg	0.72 s	0.001 s	4584 bit
q_{Tor}	1 time	-10 deg	-8.2 deg	0.05859 deg	0.72 s	0.001 s	3558 bit
Estimate of the total processed information per second:							64 137 bit/s

References

Dhariwal, P., Hesse, C., Klimov, O., Nichol, A., Plappert, M., Radford, A., Schulman, J., Sidor, S., Wu, Y., and Zhokhov, P. Openai baselines. https://github.com/openai/baselines, 2017.

Haeufle, D. F. B., Günther, M., Wunner, G., and Schmitt, S. Quantifying control effort of biological and technical movements: An information-entropy-based approach. *Physical Review E*, 89(1):012716, 2014. doi: 10.1103/PhysRevE.89.012716.

Hill, A., Raffin, A., Ernestus, M., Traore, R., Dhariwal, P., Hesse, C., Klimov, O., Nichol, A., Plappert, M., Radford, A., Schulman, J., Sidor, S., and Wu, Y. Stable baselines. https://github.com/hill-a/stable-baselines, 2018.

Lillicrap, T. P., Hunt, J. J., Pritzel, A., Heess, N., Erez, T., Tassa, Y., Silver, D., and Wierstra, D. Continuous control with deep reinforcement learning. *arXiv preprint arXiv:1509.02971*, 2015.

Mnih, V., Badia, A. P., Mirza, M., Graves, A., Lillicrap, T., Harley, T., Silver, D., and Kavukcuoglu, K. Asynchronous methods for deep reinforcement learning. In *International conference on machine learning*, pages 1928–1937, 2016.

Park, H. W., Sreenath, K., Hurst, J. W., and Grizzle, J. W. Identification of a bipedal robot with a compliant drivetrain: Parameter estimation for control design. *IEEE Control Systems Magazine*, 31(2):63–88, 2011. doi: 10.1109/MCS.2010.939963.

Schulman, J., Levine, S., Abbeel, P., Jordan, M., and Moritz, P. Trust region policy optimization. In *International Conference on Machine Learning*, pages 1889–1897, 2015.

Schulman, J., Wolski, F., Dhariwal, P., Radford, A., and Klimov, O. Proximal policy optimization algorithms. *arXiv preprint arXiv:1707.06347*, 2017.

Shannon, C. and Weaver, W. *The Mathematical Theory of Communication*. University of Illinois Press, 1949.

Sreenath, K., Park, H.-W., Poulakakis, I., and Grizzle, J. W. A Compliant Hybrid Zero Dynamics Controller for Stable, Efficient and Fast Bipedal Walking on MABEL. *The International Journal of Robotics Research*, 30(9):1170–1193, 2011. doi: 10.1177/0278364910379882.

Stollenmaier, K., Ilg, W., and Haeufle, D. F. B. Predicting Perturbed Human Arm Movements in a Neuro-Musculoskeletal Model to Investigate the Muscular Force Response. *Frontiers in Bioengineering and Biotechnology*, 8(308), 2020. doi: 10.3389/fbioe.2020.00308.

Wu, Y., Mansimov, E., Grosse, R. B., Liao, S., and Ba, J. Scalable trust-region method for deep reinforcement learning using kronecker-factored approximation. In *Advances in neural information processing systems*, pages 5279–5288, 2017.
